# Indoor Radon Exposure in Italian Schools

**DOI:** 10.3390/ijerph15040749

**Published:** 2018-04-13

**Authors:** Antonio Azara, Marco Dettori, Paolo Castiglia, Andrea Piana, Paolo Durando, Valentina Parodi, Giovanni Salis, Laura Saderi, Giovanni Sotgiu

**Affiliations:** 1 Department of Medical, Surgical and Experimental Sciences, University of Sassari, Via P. Manzella, 07100 Sassari, Italy; azara@uniss.it (A.A.); castigli@uniss.it (P.C.); piana@uniss.it (A.P.); lsaderi@uniss.it (L.S.); gsotgiu@uniss.it (G.S.); 2 Department of Health Sciences, University of Genova, Piazza Manin, 16122 Genova, Italy; durando@unige.it (P.D.); valentina.parodi@unige.it (V.P.); 3 ASSL Nuoro, ATS Sardegna, Via Monsignor Cogoni, 08100 Nuoro, Italy; giovannisebastiano.salis@atssardegna.it

**Keywords:** radon, school, schoolrooms, indoor air quality, Italy

## Abstract

Background: The aim of the study was to assess radon concentration in schoolrooms in a city located in the midwest of Italy. Methods: A two-phase environmental study was carried out in 19 school buildings of 16 primary, secondary, and tertiary schools. Results: Median (interquartile range—IQR) indoor radon concentration in schoolrooms was 91.6 (45.0–140.3) Bq/m^3^. The highest (median 952.8 Bq/m^3^) radon concentration was found in one (3.6%) classroom, located in a building of a primary school whose median concentration was 185 Bq/m^3^. Radon concentration was significantly correlated with the number of students and teachers, foundation wall construction material, and with the absence of underground floors. A geopedological survey was performed close to the building with highest radon level, showing the presence of granite and tonalithic granodiorite in the soil. Conclusions: Radon levels should be routinely assessed where individuals live or work. Schools are susceptible targets, because of childhood stay and the long daily stay of occupants. Low-cost interventions, such as implementation of natural air ventilation and school maintenance, can reduce radon levels, limiting individual exposure.

## 1. Introduction

Radon (^222^Rn) is a chemically inert, naturally occurring radioactive gas, which is part of the ^238^Uranium decay series.

In 1998, the International Agency for Cancer Research classified radon as a proven human carcinogen [1]. In 2009, the World Health Organization (WHO) recognized its role as the second leading cause of lung cancer worldwide and the primary one among people who have never smoked [2].

Nowadays, radon represents a serious public health concern owing to its accumulation in dwellings and workplaces. Because the majority of people routinely stay in indoor settings, high levels of radon can significantly increase their lung cancer risk [3]. It has been found that lung cancer occurrence mostly depends on three factors: level of radon, duration of exposure, and individual smoking habits [4].

A safe radon exposure level is currently unknown [3]. However, low concentrations can increase lung cancer risk, particularly when susceptible individuals are exposed [5].

Radon can exceed acceptable levels in confined air places because of specific climate conditions, building materials, natural and/or artificial ventilation, and the amount of uranium in the underlying rocks and soils [6,7,8].

Its indoor concentration can be assessed through long- and short-term measurements [9]. Although a long-term measurement can provide more reliable average levels, a short-term study is the most frequent and rapid approach [10].

In 1990, the European Commission (EC) issued the 90/143/Euratom Recommendation [11], including the annual average of indoor radon concentrations (200 Bq/m^3^ and 400 Bq/m^3^ in existing and new dwellings, respectively). In cases of high levels, interventions should be immediately implemented. Recently, the EC issued the 2013/59/Euratom Directive [12], in which the general reference value was set to 300 Bq/m^3^. With the 2013/59/Euratom Directive, the obligation was introduced for member countries to periodically prepare and update a Radon National Plan, which consists of a multi-annual plan to implement the set of actions necessary to reduce the risk of lung cancer associated with radon exposure. The national radon protection strategy should be straightforward and realistic and be considered in conjunction with other public health policies, such as energy saving, non-smoking, and indoor air quality. It should address exposures in new and existing buildings, aiming to reduce the overall exposure of the general population and the highest individual exposures.

To help guide action, authorities should set a national reference level for indoor radon levels.

The International Commission on Radiological Protection (ICRP) recommends setting a national reference level as low as reasonably achievable in the range of 100–300 Bq/m^3^. The WHO guidance is basically the same: a national reference level of 100 Bq/m^3^ is recommended, and wherever this is not possible, the chosen level should not exceed 300 Bq/m^3^.

In Italy, the Legislative Decree 241/2000 stated that 500 Bq/m^3^ is the highest acceptable radon concentration in workplaces [13].

The Italian Ministry of Health prepared the Radon National Plan, in which it was highlighted that schools are priority settings because of the daily exposure of students, teachers, and administrative staff, as well as because of building-related characteristics (e.g., schoolrooms mainly located on the ground level, construction materials—stone granite—of the walls and the floor, and poor natural and mechanical air ventilation) [5,14].

Sardinia is an Italian radon-prone area with a higher average radon level (60–80 Bq/m^3^) [14]. The island basement is made of metamorphites and granitic plutonites, which are sedimentary and magmatic rocks containing radioactive elements [15]; outcrops are mainly located in the eastern and central–northern regions.

Although the geopedological condition of Sardinia indirectly underscores the risk of exposure to high radon concentrations, a systematic assessment of radon levels is missing. No previous studies on radon levels have been carried out in the past, particularly in high-risk population groups, such as school children.

We performed the first scientific research on radon levels in Sardinian schools to fill the abovementioned gap.

On this basis, the aim of the present study was to assess radon concentrations in schoolrooms located in Nuoro, a northeastern Sardinian city in Italy.

## 2. Materials and Methods

This study was not approved by any ethical committees. Italian law does not require a mandatory approval for this type of environmental study.

### 2.1. Study Design

The study was carried out from 1 March 2010 to 31 July 2010 in 19 (100%) school buildings belonging to 16 primary, secondary, and tertiary schools (100.0%) located in Nuoro, Italy. A first phase was conducted to detect baseline concentrations of radon in schoolrooms. In cases of the highest concentrations, a new assessment was performed to confirm exposure to the highest radon concentrations.

All classrooms on the ground floor of the selected buildings were recruited for measurement owing to their high exposure to radon sources (direct contact with the soil and, thus, with a natural radon source).

### 2.2. Setting

Nuoro is a city in the northeastern area of Sardinia, Italy, whose population was 36,740 inhabitants in 2010 (17,464 males; 5332 children and adolescents aged between 5 and 19 years). The population of students recorded in 2010 was 4804.

A radon assessment was carried out in 19 school buildings. The study protocol described the collection of variables associated with indoor radon levels (i.e., construction date, dampness marks, restoration of the underground structural elements, and foundation walls materials) [5,8,16].

72-h radon measurements were performed in the classrooms according to the United States (U.S.) Environmental Protection Agency (EPA) “Radon Measurement in School” guidelines [10] during days of routine activity, keeping doors and/or windows closed to record the highest radon levels.

### 2.3. Radon Concentration Measurement

Investigators adopted a Continuous Radon Monitor (CRM) testing device (Model 1027, Sun Nuclear Corporation, Melbourne, FL, USA). The detector is US EPA quality tested, National Environmental Health Association National Radon Proficiency Program listed, and National Radon Safety Board approved for use in real estate transaction testing.

The device performed measurements every 60 min (i.e., 24 detections daily). A supervisor of the device activity was selected.

### 2.4. Statistical Analysis

An ad hoc electronic form was prepared to collect the main environmental variables using Excel (Microsoft Office, Microsoft Corporation, Redmond, WA, USA). Qualitative and normally distributed quantitative variables were summarized with absolute (relative) frequencies and means (standard deviations, SD), respectively. The correlation between qualitative and quantitative variables was performed using the Spearman correlation method. A two-tailed *p*-value less than 0.05 was considered statistically significant. The statistical computations were performed using STATA 15 (StatCorp., Austin, TX, USA).

## 3. Results

### 3.1. Buildings Characteristics

Radon measurements were obtained in 28 schoolrooms of 19 different buildings (Table 1).

All the buildings were built after the 1900s; in particular, 46.4% were constructed between 1900 and 1950, 32.1% between 1951 and 1964, 7.1% between 1965 and 1979, and 14.3% after 1980.

The selected classrooms hosted 603 students aged between 5 and 19 years (median (interquartile range—IQR) = 19 (16–20) students per classroom). The majority of the classrooms (92.9%) hosted students and teachers in the morning. The rooms had doors or windows closed during radon detection.

All the floor pavements were tiled (100%). A total of 85.7% of the foundation walls were made of stone, and 89.3% were in direct contact with the ground without any air cavity. Overall, 20% of the buildings were renovated in the underground floor.

The classrooms did not show water infiltrations. One room (radon median = 48.1 Bq/m^3^) showed dampness patches.

Forced-air replacement devices and/or vacuum systems were not installed in any of the classrooms; conversely, all the rooms had heating systems.

### 3.2. Radon Baseline Detection

The median (IQR) indoor radon concentration in the schoolrooms was 91.6 (45.0–140.3) Bq/m^3^.

**Figure 1:**
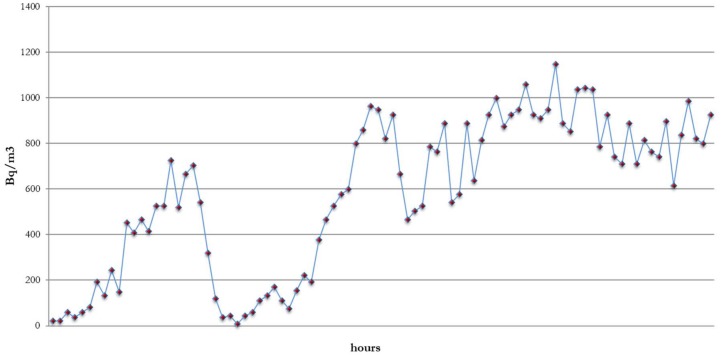
Radon concentration during the 72-h detection in Room number 3.

The highest radon (median 952.8 Bq/m^3^) concentration was found in one (3.6%) classroom (Room number 3), located in a building (primary school) whose median concentration was 185 Bq/m^3^. A second 72-h monitoring showed a median level of 651.2 Bq/m^3^, with the highest concentration (1147 Bq/m^3^) at 9:00 a.m. of the third day (Figure 1). Two other measurements were conducted after increased natural ventilation (door and windows open during break sessions and before and after daily activities: median 72-h concentrations 74 and 244.2 Bq/m^3^, respectively).

**Figure 2:**
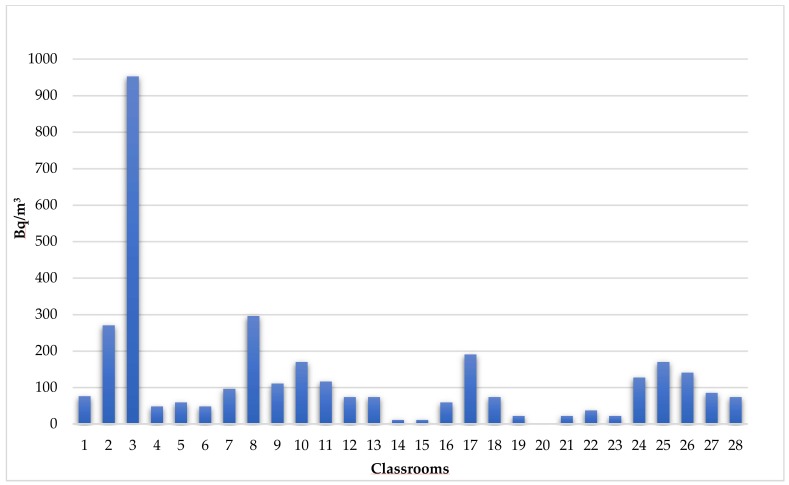
Median radon levels detected in 28 selected classrooms.

The other classrooms showed levels within the highest average levels recommended by Italian law (i.e., <500 Bq/m^3^) (Figure 2).

**Figure 3:**
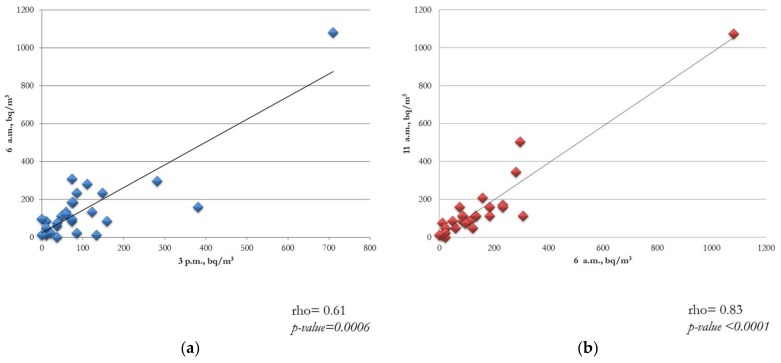
Correlation between radon concentrations and temporal periods (**a**) time-frame from 3:00 p.m. to 6:00 a.m.; and (**b**) from 6:00 a.m. to 11:00 a.m.

Figure 3a,b describes radon detections in all the 28 selected classrooms, showing how radon increased from 3.00 p.m. (at the end of the activities) until 6:00 a.m. Another temporal increase was observed between 10:30 a.m. and 11:00 a.m., during the coffee break. The lowest levels were found at approximately 2:00 p.m. Radon concentrations were significantly correlated with some temporal periods (i.e., from 3:00 p.m. to 6:00 a.m. (*r* = 0.61; *p*-value = 0.0006) and from 6:00 a.m. to 11:00 a.m. (*r* = 0.83; *p*-value <0.0001)).

Radon concentration was significantly correlated with the number of students and teachers hosted in the classrooms (*r* = −0.55; *p*-value = 0.003) and the building construction period (*r* = −0.49; *p*-value = 0.009) (Table 2). Moreover, a significant positive correlation was found between radon concentration and the foundation wall materials. Additionally, radon levels were positively correlated with the absence of underground floors.

### 3.3. Geological Evaluation

Simultaneously, a geopedological survey was carried out in areas close to the building where the highest radon levels were found. On the whole, rocks outcropping the area were mainly characterized by unaltered, even if strongly fractured, granite and tonalithic granodiorite; in some areas, however, more altered phases were observed.

## 4. Discussion

Radon levels can be key in the pathogenesis of several medical conditions, particularly cancers. Our study was aimed to assess radon concentration in 28 schoolrooms of 19 different buildings located in Nuoro, Italy.

We found that the majority of the classrooms had radon levels within the recommended highest average value, <500 Bq/m^3^, with the exception of one room, which showed the highest concentration (1147 Bq/m^3^). Other Italian authors have highlighted the crucial role played by immediate interventions aimed at reducing gas levels to decrease health risks [17]; on this basis, we recommended that school authorities act promptly to increase natural ventilation before and during school activities.

Our findings confirmed the variability of radon concentrations, which could significantly increase overnight and during coffee breaks when classrooms were closed. In particular, this result was similar to those of Bochicchio et al. and Branco et al., who described high radon concentration variability in association with different room characteristics and behaviors of teachers and students, such as keeping windows and doors closed [5,18]. On this basis, students and teachers were strongly recommended to open windows and/or doors during their absence and before starting their educational activities.

Furthermore, we found that the gas levels significantly increased with foundation stone walls and in the absence of any underground floor, as pointed out by WHO and recently argued by Baeza et al. [2,8]. They confirmed the risk of higher radon concentrations with stone- (e.g., granite) and wood-based construction materials in old dwellings and the decrease of environmental radon exposure in new buildings, whose ground floors are characterized by boulder beds and a polythene sheets [8]. In contrast, school buildings constructed after the 1980s with elevated demographic density (i.e., greater number of students and teachers in the classrooms) showed lower radon concentrations. These results confirmed the findings of previous studies [8,18] that determined that higher overnight radon concentrations could be explained by poor air renovation, whereas lower daily concentrations depended on a higher air turnover through natural (windows opened by students and/or teachers) or mechanical ventilation.

Contextually, a geopedological survey proved that areas close to the building with the highest radon levels were made of granite and tonalithic granodiorite. As underscored by Buondonno et al. and Trevisi et al. [19,20], sandy soils are characterized by huge permeability and porosity, thus promoting gas leaking processes, particularly when differences of temperatures between night and day are significant. Uranium in the rocks, easily leached by rains enriching the soils and deep waters, can be transformed to radon through a decay process.

The results on radon level variability in classrooms highlight the key role of monitoring activities on indoor air quality and, consequently, on the health of occupants. Based on the increasing body of evidence on the relationship between radon levels and chronic diseases, radon concentrations should be routinely monitored where individuals live or work. In particular, as underscored by several studies [4,5,6,21,22,23,24,25,26,27,28], attention should be focused on schools, where students, teachers, and other personnel spend numerous hours daily. Apart from the long exposure to elevated radon concentrations, several concerns should be raised regarding the potential high vulnerability of some population groups, such as children.

Passive monitoring systems are used in the majority of the cases; however, continuous monitoring devices can provide long-term assessment and thereby, an improved diagnostic accuracy. Short-term surveys represent a more rapid means to monitor radon concentrations, suggesting when interventions should be implemented, as recommended by the U.S. EPA [10,29]. However, in cases where a high variability of radon levels is detected, a different strategy based on long-term monitoring systems could provide more detailed and accurate information.

## 5. Conclusions

Our study demonstrated a risk of exposure to high radon concentrations in Italian schools. Comprehensive monitoring activities should be implemented to better describe this environmental hazard. Interventions should be planned, depending on architectural elements, soil characteristics, and human behaviors. Although the majority of Italian educational buildings are of historic interest, immediate solutions should be adopted to preserve them from deterioration and to reduce the health risk of their occupants. Actions should take into consideration both ground permeability and soil characteristics, particularly in radon-prone areas. Furthermore, education is needed to improve human behavior, focusing on the importance of natural ventilation.

Our study shows several limitations. The study was carried out in an Italian city located in a specific geographical context. However, findings can be generalizable to similar areas having buildings characteristics comparable to those described in Nuoro. The short-term assessment does not provide a comprehensive and detailed kinetic of the radon concentrations. However, the relationship with some variables could be helpful to implement rapid interventions aimed at reducing radon levels.

Low-cost interventions, such as the improvement of natural air ventilation, increased under-floor ventilation that prevents the passage of radon from the basement into living rooms, sealed floors and walls, and education on air ventilation, can reduce indoor radon levels, limiting individual exposure.

## Figures and Tables

**Table 1:** Radon measurements in 28 schoolrooms of 19 different buildings. Descriptive analysis.

Descriptive Analysis Results		
Median (IQR) no. students/teacher		19 (16–20)
Construction period, n (%)	<1900	0/28 (0.0)
	1900–1950	13/28 (46.4)
	1951–1964	9/28 (32.1)
	1965–1979	2/28 (7.1)
	≥1980	5/28 (14.3)
Underground renovation, n (%)		3/15 (20.0)
Foundation wall material, n (%)	Wood	0 (0.0)
	Cement	4/28 (14.3)
	Stone	24/28 (85.7)
Flooring, n (%)	Cement	0 (0.0)
	Floor tiles	2/28 (100.0)
	Wood	0 (0.0)
	Rubber	0 (0.0)
	Linoleum	0 (0.0)
First floor above underground floor, n (%)		2/28 (7.1)
First floor without any underground floor, n (%)		25/28 (89.3)
External entrance doors, n (%)		9/28 (32.1)
Usage of the doors, n (%)		9/9 (100.0)
Windows, n (%)		28/28 (100.0)
Foundation walls adjacent to a cavity wall, n (%)		0/27 (0.0)
Foundation walls adjacent to an air cavity, n (%)		0/27 (0.0)
Foundation walls adjacent to ground, n (%)		27/27 (100.0)
Foundation rocky walls, n (%)		0/27 (0.0)
Water infiltration, n (%)		0/27 (0.0)
Condensate/Humidity, n (%)		1/27 (3.7)
Heating system, n (%)		27/27 (100.0)
Afternoon class, n (%)		2/28 (7.1)
Forced air exchange, n (%)		0/28 (0.0)
Vacuum system, n (%)		0/28 (0.0)
Exposed piping, n (%)		10/28 (35.7)
Piping, n (%)	In the floor	0/20 (0.0)
	In the side wall	28/28 (100.0)
Floor and wall cracks and fissures of undergrounds rooms, n (%)		0/28 (0.0)
Previous Radon measurement, n (%)		1/28 (3.6)

**Table 2:** Spearman correlation analysis between environmental variables and radon concentration levels.

Variables	No. Students/Teacher	Construction Period	Foundation Wall Material	Condensate/Humidity	First Floor without Underground Floor
Construction period	0.18				
*p*-value	0.368				
Foundation wall material	−0.52	−0.62			
*p*-value	0.005	0.001			
Condensate/Humidity	0.13	−0.20	0.08		
*p*-value	0.524	0.312	0.685		
First floor without underground floor	−0.31	−0.08	0.28	0.06	
*p*-value	0.111	0.700	0.157	0.784	
Radon concentration, Bq/m^3^	−0.55	−0.49	0.44	−0.13	0.38
*p*-value	0.003	0.009	0.021	0.532	0.050
